# Correlation of Homeostatic Model Assessment-Insulin Resistance, Anti-Mullerian Hormone, and BMI in the Characterization of Polycystic Ovary Syndrome

**DOI:** 10.7759/cureus.16047

**Published:** 2021-06-30

**Authors:** Anupama Bahadur, Neha Verma, Rajlaxmi Mundhra, Latika Chawla, Megha Ajmani, Modalavalasa Swetha Sri, Shivaani Arora

**Affiliations:** 1 Obstetrics and Gynecology, All India Institute of Medical Sciences, Rishikesh, IND

**Keywords:** reproductive age group, pcos, homa-ir, bmi, amh

## Abstract

Objective: To evaluate the correlation of homeostatic model assessment-insulin resistance (HOMA-IR), anti-Mullerian hormone (AMH), and BMI and to compare their values across the different phenotypes in polycystic ovary syndrome (PCOS) women of the reproductive age group.

Study design: A total of 307 PCOS-diagnosed women were included in the study and further classified in different phenotypes. BMI, HOMA-IR, and serum AMH values were noted and their association with different phenotypes was seen. The correlation of these variables was also noted.

Results: Phenotype D was the most common phenotype followed by type A, type B, and type C. A total of 265 women had an AMH value of ≥4 mg/ml with the highest value in phenotype A followed by D, C, and B. HOMA-IR and BMI values did not vary significantly among different phenotypes. HOMA-IR and BMI had a statistically significant positive correlation and serum AMH was negatively correlated with HOMA-IR, but no significant correlation was seen between serum AMH and BMI. The biochemical parameters like luteinizing hormone (LH), follicle-stimulating hormone (FSH), LH: FSH ratio, and serum testosterone showed no correlation with phenotypes or any other clinical parameter.

Conclusion: HOMA-IR and BMI showed a statistically significant positive correlation indicating the need for lifestyle modification and weight reduction in PCOS women, which can further help in decreasing insulin resistance. A strong correlation of serum AMH levels and phenotypes shows the importance of serum AMH levels for classifying different PCOS phenotypes.

## Introduction

Polycystic ovary syndrome is one of the most common endocrine disorders among the reproductive age group women with a prevalence of 6-10% [1]. It is characterized by the presence of varying features like hirsutism, acne, menstrual irregularities, infertility, insulin resistance, impaired glucose tolerance or diabetes mellitus, metabolic syndrome, dyslipidemia, cardiovascular disease, psychosocial problems like depression, anxiety, and poor quality of life. The diagnostic criteria of PCOS have changed with time, according to expansion in our understanding of reproductive medicine and due to advances in medical technology.

Rotterdam PCOS diagnostic criteria consist of the presence of two out of the following three parameters: oligo-ovulation/anovulation, clinic, and/or laboratory signs of hyperandrogenism, and polycystic ovarian morphology at ultrasonography (≥ 20 follicles of 2-9 mm), after excluding other endocrinopathies having a similar clinical presentation [2,3].

PCOS women can be classified into four phenotypes: phenotype A (full‑blown syndrome PCOS: hyperandrogenism; HA + ovulatory dysfunction {OD} + PCO), phenotype B (HA+OD), phenotype C (ovulatory PCOS: HA+PCO), and phenotype D (non‑hyperandrogenic PCOS: OD+PCO).

Anti-Mullerian hormone (AMH) also known as Mullerian inhibiting substance (MIS) is a member of the transforming growth factor-beta (TGF-β) superfamily [4]. It is produced by granulosa cells with the greatest expression seen in granulosa cells of follicles measuring less than 4 mm (preantral follicles and antral follicles). In PCOS women, there is an excessive amount of AMH, which is explained by increased follicles in the preantral and antral stages. Lately, it has been identified as another potential marker for diagnosing PCOS as studies have also demonstrated that its production is increased severalfold in anovulatory PCOS and normo-ovulatory PCOS women when compared to “normal” ovaries, suggesting a dysregulation of the granulosa cells.

Apart from being an ovulatory disorder, PCOS is also a metabolic disorder. Insulin resistance (IR) with subsequent compensatory hyperinsulinemia are closely related to its pathogenicity and comorbidities and can be worsened by the coexistence of obesity, which affects almost 50% of PCOS women [5]. Recent evidence has suggested that adiposity be an additional component to IR in obese PCOS [6-9]. However, it is still not a defining criterion for PCOS. Moreover, PCOS itself has been shown to confer a risk for IR, beyond that caused by obesity alone [10]. Hyperinsulinemia has been shown to promote androgen synthesis independently of gonadotropins thereby contributing to the androgenic milieu in ovaries resulting in the hyperandrogenic phenotype of PCOS [1]. Homeostatic model assessment-insulin resistance (HOMA-IR), a marker for IR, is less invasive compared to gold standard glucose clamp methods and is shown to have a linear correlation with the glucose clamp in various studies [11]. Many studies have used 2.5 as the cut-off value [1]. It has been also proposed that IR might also be related to serum anti-Mullerian hormone levels.

Obesity, high AMH, and insulin resistance (IR) are shown to be strongly associated with PCOS. Hence, this study was conducted to study the correlation of BMI, HOMA-IR, and AMH and to compare their values across different phenotypes in women with PCOS of the reproductive age group. 

## Materials and methods

This cross-sectional, observational study was conducted in the Department of Obstetrics and Gynecology of a tertiary care hospital in the hilly terrain of India. The women presenting with complaints of irregular menstruation, hirsutism, acne, or infertility between January 2019 and March 2020 were reviewed and Rotterdam criteria were used for diagnosis of PCOS. The baseline demographic variables of all the selected participants were noted. Clinical history was taken and oligomenorrhea was defined as the menstrual cycle of > 35days or < 8 cycles per year. Hyperandrogenism was scored according to the modified Ferriman Gallwey score. The scores < 4 indicate mild hirsutism, scores 4-7 indicate moderate hirsutism, and scores ≥ 8 indicate severe hirsutism. The global acne score was used to see acne. BMI was also noted. A gynecological examination was performed, and USG was done. Polycystic ovarian morphology (PCOM) was defined by the presence of ≥ 20 follicles per ovary and ovarian volume ≥ 10 ml in either ovary. Blood investigations including serum thyroid-stimulating hormone (TSH), serum prolactin, day 2/3 luteinizing hormone (LH) and follicle-stimulating hormone (FSH) levels, serum testosterone, serum AMH, fasting blood sugar and fasting insulin were done. Serum AMH level was done using an ultrasensitive anti-Mullerian hormone/Mullerian inhibiting substance (AMH/MIS) enzyme-linked immunosorbent assay (ELISA) kit, which was a three-step sandwich-type immunoassay. IR was measured by means of homeostatic model assessment of IR (HOMA-IR = fasting blood sugar (mg/dl) x fasting insulin mIU/l/405) with a cut-off value of ≥ 2.5. After confirming the diagnosis of PCOS, the recruited participants in our study were further classified into the four phenotypes.

The outcome of our study was to see the correlation of serum AMH, BMI, and IR and to compare their values across different phenotypes in PCOS women of the reproductive age group.

The sample size was calculated based on a previous study done by Gupta et al., which reported the prevalence of Phenotype D in PCOS as 24.6% [12]. The sample size was calculated using formula N = (z1 - α/2)­2 x p x (1 - p)/ δ2, with p=0.246, Type I error (α) =5% and Precision (δ) =5%. Thus, the proposed sample size of the study was 289 PCOS women.

## Results

In the present study comprising of 307 participants, maximum patients (150, 48.9%) belonged to phenotype D, the second most common was phenotype A (114, 37.1%) followed by type B (25, 8.1%) and type C (18, 5.9%). The baseline characteristics of the participants are shown in Table 1.

**Table 1:** Baseline characteristics IQR: interquartile range; LH: luteinizing hormone; FSH: follicle-stimulating hormone; HOMA-IR: homeostatic model assessment-insulin resistance; AMH: anti-Mullerian hormone

​ Baseline Characteristics	Mean ± SD || Median (IQR) (n=307)
Age (Years)	23.56 ± 4.30 || 23.00 (6.00)
BMI (kg/m^2^)	26.41 ± 4.55 || 26.22 (4.98)
HOMA-IR	3.75 ± 2.51 || 2.98 (3.06)
Serum AMH (ng/ml)	6.94 ± 3.65 || 5.60 (4.81)
LH (mIU/ml)	8.26 ± 5.13 || 7.15 (5.59)
FSH (mIU/ml)	5.72 ± 1.80 || 5.50 (2.05)
Fasting Insulin (mIU/ml)	16.33 ± 9.24 || 14.60 (12.33)
Fasting Blood Sugar (mg/dl)	89.47 ± 13.09 || 88.00 (16.70)

**Figure 1:**
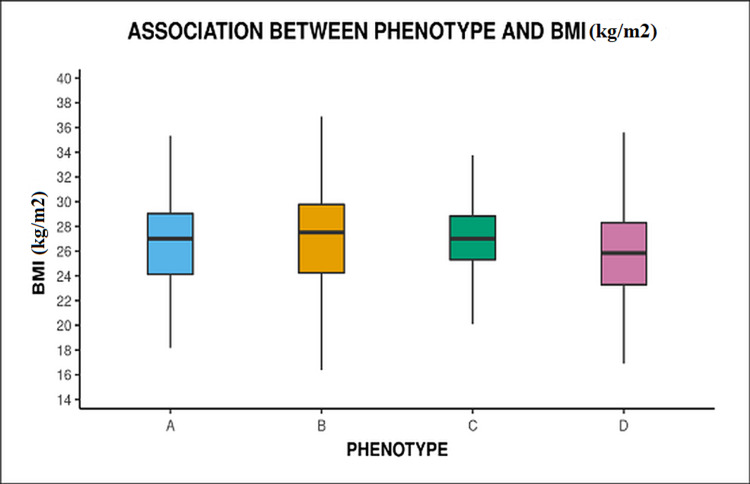
Box and whisker plot showing association of BMI with different phenotype

The mean BMI in all phenotypes was more than 25 kg/m^2^, as well as when the study population was distributed in different BMI categories then more than half (209, 68.08%) of the participants were obese, i.e., BMI ≥25 kg/m^2^. However, no significant difference was seen in the mean BMI across different phenotypes (p = 0.293) (Figure 1). In our study 180 (58.6%) participants had a HOMA-IR value of ≥ 2.5.

**Figure 2:**
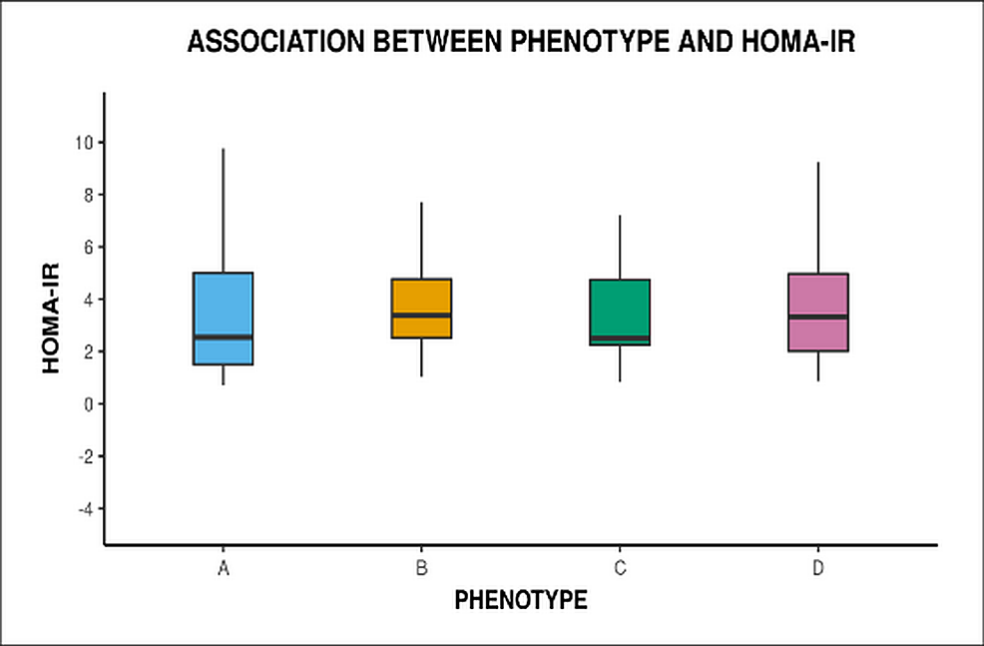
Box and whisker plot showing association of HOMA-IR with different phenotype HOMA-IR: homeostatic model assessment-insulin resistance

The distribution of insulin resistance was found almost the same and no significant difference was found in the median HOMA-IR levels between the phenotypes (Figure 2). Our study found that 265 (86.3%) out of total women had AMH levels of more than 4 ng/ml with a median value of 5.6 ng/ml.

**Figure 3:**
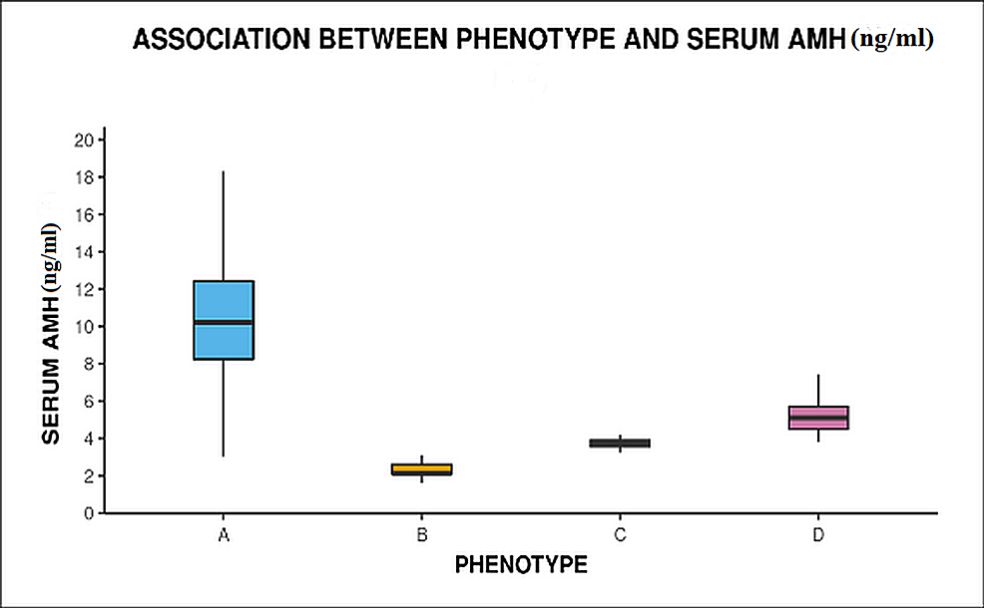
Box and whisker plot showing association of serum AMH with different phenotype AMH: anti-Mullerian hormone

On further analysis, it was found that values of serum AMH levels significantly varied between different phenotypes (p < 0.001) (Figure 3).

The mean serum AMH level was 10.51 ng/ml, significantly higher compared with other phenotypes (p < 0.001) as well as mean serum AMH value of phenotype B was within the normal range (2.6 ng/ml) (Table 2). There was no difference in baseline LH and LH/FSH ratio in different phenotype groups (Table 2).

**Table 2:** Clinical and biochemical parameters in different phenotypes LH: luteinizing hormone; FSH: follicle-stimulating hormone; HOMA-IR: homeostatic model assessment-insulin resistance; AMH: anti-Mullerian hormone

Parameters	Phenotype A n=114 Mean ± SD	Phenotype B n=25 Mean ± SD	Phenotype C n=18 Mean ± SD	Phenotype D n=150 Mean ± SD	p-Value
BMI (kg/m^2^)	26.89 ± 4.62	26.96 ± 5.43	26.90 ± 5.17	25.90 ± 4.24	0.293
LH (mIU/ml)	7.79 ± 4.12	8.03 ± 3.45	8.68 ± 5.60	8.61 ± 5.95	0.900
FSH (mIU/ml)	5.70 ± 1.64	5.57 ± 1.73	5.35 ± 1.03	5.81 ± 1.99	0.828
LH:FSH Ratio	1.41 ± 0.73	1.71 ± 1.30	1.72 ± 1.24	1.56 ± 1.00	0.791
Fasting Blood Sugar (mg/dl)	88.97 ± 12.57	88.36 ± 12.20	86.78 ± 14.24	90.36 ± 13.52	0.638
Fasting Insulin (µIU/ml)	15.40 ± 9.48	17.36 ± 8.06	17.54 ± 10.46	16.71 ± 9.12	0.335
HOMA-IR	3.52 ± 2.52	3.88 ± 2.14	3.95 ± 2.90	3.87 ± 2.51	0.392
Serum AMH (ng/ml)	10.51 ± 3.31	2.60 ± 1.42	3.60 ± 0.51	5.34 ± 1.37	<0.001

**Figure 4:**
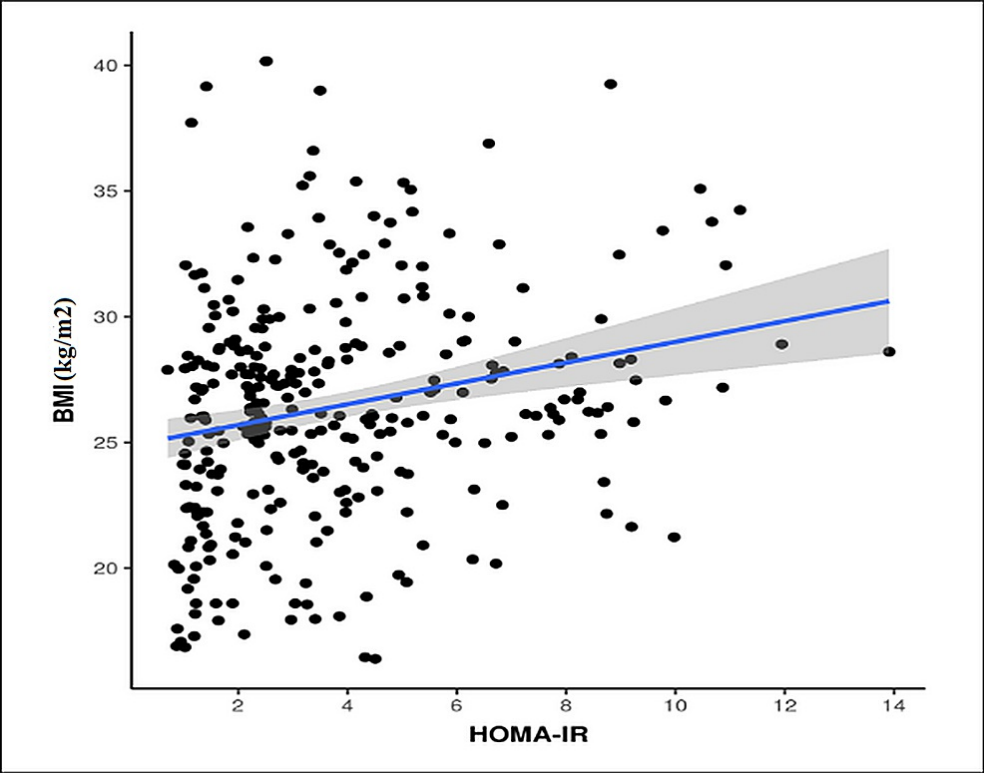
Correlation between BMI and HOMA-IR (p<0.001) HOMA-IR: homeostatic model assessment-insulin resistance

A statistically significant positive trend in correlation was observed between BMI and HOMA-IR (p < 0.001) (Figure 4). On further evaluation, the mean value of BMI was higher (27.12 kg/m^2^) in patients with insulin resistance as compared to patients without insulin resistance.

**Figure 5:**
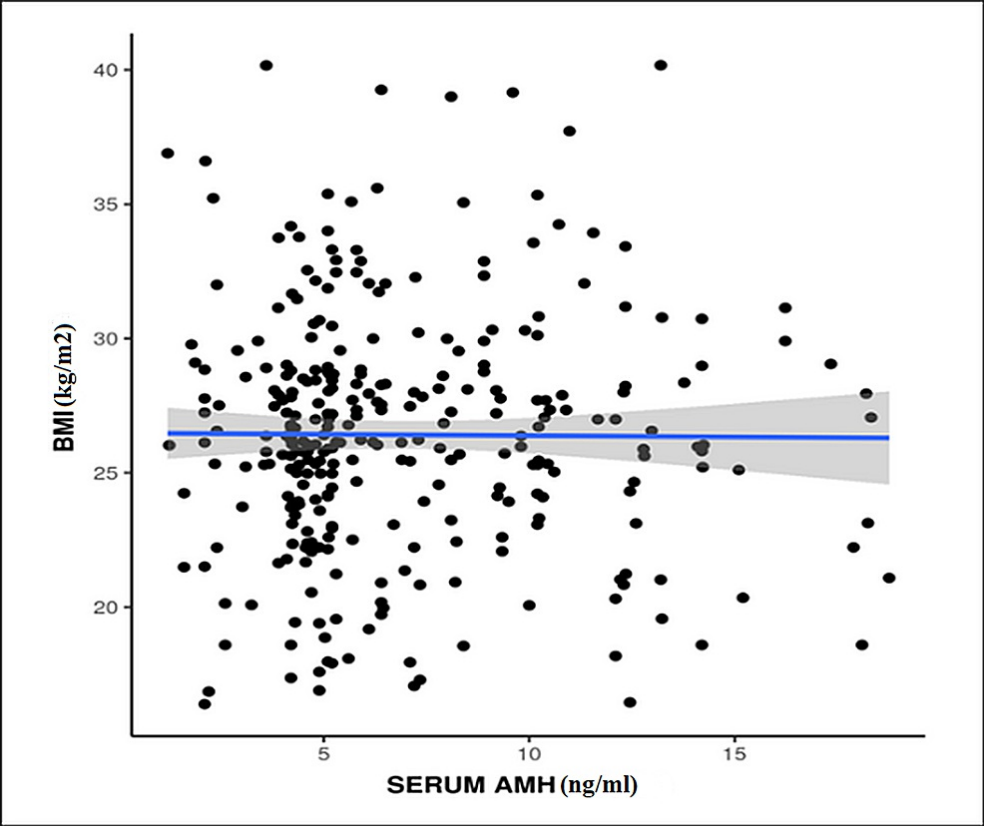
Correlation between BMI and serum AMH (p = 0.749) AMH: anti-Mullerian hormone

A weak positive correlation was seen between BMI and AMH, which was not statistically significant (p = 0.749) (Figure 5). Similarly, no significant difference was seen between different grades of BMI in terms of serum AMH (ng/mL) (p = 0.781).

**Figure 6:**
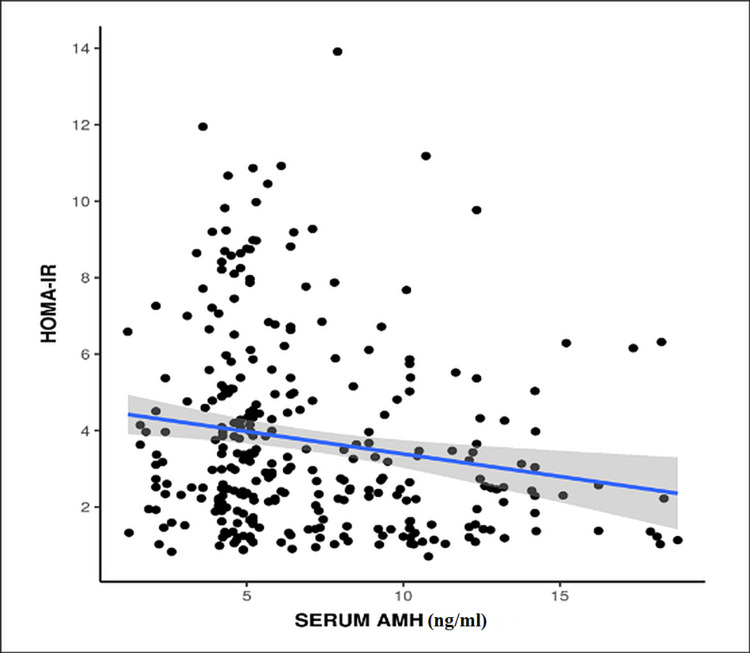
Correlation between HOMA-IR and serum AMH (p = 0.002) HOMA-IR: homeostatic model assessment-insulin resistance; AMH: anti-Mullerian hormone

After comparing serum HOMA-IR and serum AMH, a weak negative correlation was found between the two that was statistically significant (p = 0.002) (Figure 6).

## Discussion

The most common phenotype in our study was type D followed by type A, type B, and type C. Similar findings were found in a study by Sova et al. and Zhang et al. [13,14]. Contrary to this, phenotype A was the most prevalent in studies conducted by Polak et al., Wiweko et al., Romualdi et al., and Gupta et al. [12,15-17]. This variable distribution of different phenotypes can be attributed to variable geographic locations, ethnicity, and other social and cultural practices. The plausible explanation for a maximum number of phenotype D in our study is due to the low occurrence of hyperandrogenism in the Asian population [18,19].

In our study, the mean BMI was 26.41 ± 4.55 kg/m^2^ and more than half (209, 68.07%) of the participants were obese, i.e., BMI ≥ 25 kg/m^2^ with maximum (149) women in obese category I (25-29.9 kg/m^2^). A systematic review and meta-analysis done by Lim et al. based on 21 studies reported 61% pooled prevalence for overweight or obesity in women with PCOS compared with controls [20].

But, in our study, there was no significant difference (p=0.293) in mean BMI between different phenotypes, supporting findings observed in a study by Wiweko et al. [16]. Contrary to this in the study conducted by Gupta et al., a significant difference in mean BMI between the phenotypes (p=0.003) was noted, and on the comparison, the mean BMI in phenotype B was significantly higher than C and D [12].

The serum anti-Mullerian hormone value of the participants ranged from 1.2 to 18.76 ng/ml and 265 patients out of 307 had an AMH value of ≥ 4 mg/ml and levels significantly (<0.001) varied between different phenotypes. The median AMH value was found the highest in phenotype A followed by phenotype D, C, and B. This finding may be suggestive of AMH being a marker of severity of disease as type A has been considered the most severe of the lot which comprises all three features of PCOS.

The prevalence of Insulin resistance in our study (58.6%) correlated with previous studies varying between 44% and 70%. It can be considered as a surrogate marker for severity of the condition and being higher in more severe phenotypes, moreover, finding this correlation may help in targeted therapy in different phenotype groups. However, in our study distribution of insulin resistance in terms of HOMA-IR was found almost the same and no significant difference was found in median HOMA-IR levels between the phenotypes.

Our study showed a positive correlation between BMI and HOMA-IR, which was similar to various other studies, thus it can be proposed that diet and lifestyle modification along with a weight reduction can improve the symptoms of PCOS by reducing insulin resistance, which plays an important role in its pathogenesis [1,21,22].

There have been conflicting results on the correlation between BMI and serum AMH, some showing negative correlation [13,23] while others have shown no correlation [1,12,24]. Our study showed a weak positive correlation that was statistically not significant (p = 0.749). Thus, on the basis of such conflicting results, it is not clear whether BMI can be used to determine the levels of serum AMH levels clinically. Therefore, we require further studies with a larger sample size to clarify the association and pathophysiology between BMI and serum AMH.

In our study, after comparing HOMA-IR and serum AMH, a weak negative correlation was found between the two and this correlation was found to be statistically significant (p = 0.002). On reviewing the literature, several studies have shown contradictory findings, some with positive correlation [25-28] while others have shown negative [13,19] or no correlation [1,10,12].

The strength of our study lies in the large sample size that has been used to evaluate correlations of various parameters. Apart from this, to the best of our knowledge, this is the first time to investigate these findings among different PCOS phenotypes in the reproductive age group in North India. Our study’s main limitation was the use of HOMA-IR to measure insulin resistance instead of the gold standard glucose clamp method. Moreover, additional variables affecting insulin resistance like physical activity, dietary habits were not computed in our study.

## Conclusions

Polycystic ovary syndrome is one of the most important endocrine disorders affecting reproductive-age women. Its classification in different phenotypes can be helpful in the targeted treatment of the disease. The prevalence of different phenotypes varies according to demography, ethnicity, and lifestyle. In our study, phenotype D was found to be the most prevalent of all. The results of this study indicated a strong correlation between serum AMH value and phenotype. However, no further correlation was established between other parameters like BMI and HOMA-IR. Further detailed study of the distribution of various clinical and biochemical factors among the phenotypes can be beneficial for a targeted approach to improve outcome and to prevent further complications related to the disease, as well as this correlation will give a better understanding of the disease and any change in classification can be done accordingly.

There is a positive correlation between BMI and HOMA-IR, therefore, the evaluation of IR should be done in overweight and obese women and should be encouraged for lifestyle modification. There was a weak negative correlation between HOMA-IR and AMH and no correlation between BMI and AMH. The controversies in different studies call for the need for multicentric studies, involving various demographic factors to clarify the correlation and to better understanding the disease that affects such a significant part of the female population.
